# Machine Learning‐Based Risk Stratification for Metabolic Dysfunction Severity Among Diabetic Patients With Established MASLD: Distinguishing Normal‐Weight From Overweight Individuals

**DOI:** 10.1155/jdr/4207980

**Published:** 2026-04-15

**Authors:** Yuelan Yin, Sa Ke, Yilin Liu, Xiaoling Wang, Qing Xue, Weining Sun

**Affiliations:** ^1^ Department of General Practice, The Fifth Affiliated Hospital of Sun Yat-sen University, Zhuhai, China, sysu.edu.cn; ^2^ Department of Endocrinology, Xinhua Hospital Affiliated to Shanghai Jiao Tong University School of Medicine, Shanghai, China, xinhuamed.com.cn

**Keywords:** fatty liver, lean MASLD, machine learning, metabolic dysfunction-associated steatotic liver disease (MASLD), predictive models

## Abstract

**Objective:**

To rank biochemical and clinical features that distinguish normal‐weight (BMI 18–24 kg/m^2^) from overweight diabetics already carrying a discharge diagnosis of metabolic dysfunction‐associated steatotic liver disease (MASLD), and to build a parsimonious model that can flag lean individuals at highest risk of advanced metabolic complications.

**Methods:**

This study collected a total of 3524 samples from hospitalized patients with diabetes and nonalcoholic fatty liver disease (NAFLD), with 54 NAFLD features serving as the original dataset. After data preprocessing, 2624 samples and 52 NAFLD features were screened from the original dataset to form the final dataset for model input. Among these, 1848 patients were labeled as Class 0 (BMI > 24 kg/m^2^), and 776 patients were labeled as Class 1 (BMI between 18 and 24 kg/m^2^). Data visualization and exploratory data analysis were performed using t‐SNE and heat maps. A five‐fold cross‐validation with 10 repetitions was employed for model optimization. The predictive model was evaluated using a confusion matrix. Three optimal predictive models with the smallest error were established: random forest, logistic regression, and gradient boosting. The top 30 feature variables were ultimately selected. An independent dataset containing 699 cases was used for external validation.

**Results:**

Uric acid, vitamin D, hemoglobin, and creatine kinase are the most significant features in normal‐weight diabetes patients with MASLD. Gradient boosting was considered the best model; the average area under the ROC curve (AUC) was 0.733 (95% CI: 0.7089–0.7578).

**Conclusion:**

Gradient boosting is the optimal predictive model, which can assist healthcare professionals in risk assessment and management for diabetic MASLD patients with normal BMI.

## 1. Introduction

Metabolic steatotic liver disease (metabolic dysfunction‐associated steatotic liver disease [MASLD]) is a group of diseases characterized by excessive accumulation of fat in the liver without a history of significant alcohol consumption [1, 2]. The global prevalence of metabolic‐associated fatty liver disease (MASLD) is increasing in parallel with the rise in type 2 diabetes mellitus (T2DM) and obesity [3, 4]. Cirrhosis and hepatocellular carcinoma, caused by MASLD, are also rising. Moreover, MASLD and T2DM together contribute to the development of cardiovascular disease (CVD) and chronic kidney disease (CKD), amongst other conditions [3, 5, 6]. Furthermore, an increased morbidity and mortality from liver‐related diseases and nonhepatic malignancies are observed in patients with MASLD [4, 7]. The link between MASLD and diabetes is well‐established, with numerous studies indicating that MASLD is not only more common in individuals with diabetes but also that it can independently predict the development of T2DM [8, 9]. This bidirectional relationship underscores the importance of identifying and managing MASLD in diabetic patients to prevent disease progression and related complications.

MASLD may affect and progress in both obese and lean individuals. Lean subjects are predominantly males, have a younger age at diagnosis, and are more prevalent in some geographic areas. Lean subjects can develop hepatic and extrahepatic disease in the absence of weight gain [10, 11]. These features highlight the unique nature of lean MASLD, which occurs in the normal BMI (18–24 kg/m^2^) and requires specific clinical attention. The need for a prediction model to identify normal BMI diabetic patients with MASLD arises from the fact that MASLD is typically associated with obesity, but a significant proportion of patients, particularly in Asian populations, have normal BMI [12]. These patients may be overlooked using traditional diagnostic methods that rely heavily on BMI as a screening tool. As a result, they might not receive timely interventions, leading to potential progression to more severe liver diseases, including nonalcoholic steatohepatitis (NASH), liver fibrosis, cirrhosis, and hepatocellular carcinoma [13, 14].

Machine learning (ML) demonstrates significant potential in medical diagnosis and predictive modeling. By processing large amounts of complex data and identifying potential patterns, it improves the accuracy (ACC) of disease development and outcome prediction [15, 16]. For instance, the model developed by Google DeepMind predicted acute kidney injury (AKI) 48 h earlier with an ACC of 55.8%, compared to the traditional method’s ACC of only 14.8% [17]. ML can also provide early warning through a variety of data analyses. A multicenter retrospective study in China utilized 98 laboratory tests and clinical characteristics from women with or without ovarian cancer. It employed the classification of a multiple standard decision fusion (MCF) risk prediction framework to establish a disease prediction model. The model exhibited superior and stable performance in terms of area under the ROC curve (AUC) and sensitivity, offering a low‐cost, easily accessible, and accurate auxiliary diagnostic tool for ovarian cancer [18].

In conclusion, unlike diagnostic models that require imaging‐negative controls, our ranking approach addresses a distinct clinical need: among diabetic patients already coded as MASLD, clinicians must decide who merits the most aggressive work‐up, because lean individuals can progress to fibrosis and cardiovascular events despite normal BMI. Such a model will be of important value for early detection and personalized intervention strategies, ultimately improving patient outcomes. This approach aligns with the growing body of research highlighting the importance of ML in enhancing medical diagnosis and prognosis [19].

The absence of imaging‐negative diabetic controls precludes estimation of true specificity (SPE); therefore, our model should not be used to diagnose MASLD in the general diabetic population—it is intended only for risk stratification among patients already diagnosed with MASLD.

## 2. Materials and Methods

### 2.1. Study Subjects

As a retrospective ranking study, because our database lacks imaging‐negative controls, we restricted analysis to subjects already carrying the MASLD label and modeled BMI category within this cohort. This study initially selected 3524 inpatients with both diabetes and nonalcoholic fatty liver from the Fifth Affiliated Hospital of Sun Yat‐sen University from January 2023 to December 2023 through the Haitai Hospital System as the study subjects. And 877 inpatients with diabetes and nonalcoholic fatty liver from the Fifth Affiliated Hospital of Sun Yat‐sen University from January 2024 to December 2024 through the Haitai Hospital System were selected for external validation. Inclusion criteria: ① The discharge diagnosis must meet both “diabetes” and “nonalcoholic fatty liver.” ② For patients with multiple hospitalizations, only the information from the first hospitalization of that year was extracted. Patient information, including age, gender, BMI, blood routine, liver function, kidney function, tumor markers, etc., was extracted. All inpatients signed an informed consent form and were willing to participate in this study.

We categorized BMI into binary classes (normal: 18–24 kg/m^2^ vs. overweight: ≥24 kg/m^2^) based on the following considerations: (1) Clinical actionability: The WHO Asia‐Pacific cutoff for overweight is widely used in clinical practice to trigger specific management pathways, including enhanced fibrosis screening and specialist referral. Binary classification directly supports this decision point. (2) Pathophysiological distinction: Lean MASLD and overweight MASLD represent distinct phenotypes with different metabolic profiles, natural histories, and prognostic implications. The binary split captures this clinically meaningful stratification. (3) Model interpretability and clinical utility: Binary classification aligns with actionable clinical questions (e.g., “Should this normal‐BMI patient undergo enhanced monitoring?”). Continuous BMI predictions would require arbitrary thresholding for clinical implementation, potentially introducing operator‐dependent variability. (4) Class imbalance considerations: Our dataset exhibits a 70:30 distribution (overweight:normal), which, while imbalanced, is manageable with Synthetic Minority Oversampling Technique (SMOTE). Treating BMI as continuous would introduce right‐skewness (typical of clinical populations) and heteroscedasticity, complicating model assumptions without clear clinical benefit. (5) Primary objective alignment: Our goal is risk stratification into clinically relevant groups, not precise BMI estimation. The binary approach optimizes sensitivity for identifying high‐risk lean patients, which is the primary unmet clinical need. We did not evaluate continuous BMI prediction, which may capture within‐category heterogeneity but lacks direct clinical actionability.

### 2.2. Data Preprocessing

To enhance the ACC and stability of subsequent modeling, eliminate interference factors such as noise, missing values, and outliers in the raw data, and ensure that the data meets modeling requirements, this study adopted a multistep progressive data preprocessing pipeline. The specific strategies, algorithms, and key parameter settings for each step are detailed below. A summary of the process parameters is provided in Table 1.

**Table 1:** The preprocessing steps, algorithms, and parameters executed on the original dataset and external validation set in Experiments 1 and 2.

Experiment	Data preprocessing steps	Strategy	Algorithm	Parameters
Experimental 1	1	Feature selection	—	Missing rate threshold = 60%
	2	Sample filtering	—	Missing rate threshold = 30%
			Outlier removal (IsolationForest)	Contamination = 0.02
	3	Log transformation	—	—
	4	Missing value imputation	Expectation–maximization (EM)	Iterations = 150
	5	Inverse log transformation	—	—
	6	Scaling	StandardScaler	—
	7	Oversampling	SMOTE	—
Experimental 2	1	Sample filtering	—	Missing rate threshold = 30%
	2	Log transformation	—	—
	3	Missing value imputation	IterativeImputer (EM)	max_iter = 300
	4	Inverse log transformation	—	—
	5	Scaling	StandardScaler	—

#### 2.2.1. Feature Selection

Using the feature missing rate as the core screening indicator, a threshold of 60% was set. Features with a missing rate exceeding this threshold were removed. This step effectively reduces the interference of ineffective features in modeling, decreases computational complexity, and avoids modeling bias caused by imputation of features with high missing rates.

#### 2.2.2. Sample Filtering

Following feature selection, sample filtering was conducted using dual criteria to ensure sample quality. First, with a missing rate threshold of 30%, invalid samples where the missing rate of key features exceeded this threshold were excluded. Second, outlier removal was performed for numerical features using the Isolation Forest algorithm. The outlier proportion parameter was set to contamination = 0.02, assuming that outliers account for 2% of the dataset. The algorithm automatically identified and removed these outliers to mitigate the impact of extreme values on model training.

#### 2.2.3. Missing Value Processing

Due to the presence of missing values in the dataset, features with more than 60% missing values were deleted. Features with less than 60% missing values were imputed, that is, the missing values in the data were predicted using the available data and their internal relationships.

Before applying the expectation–maximization (EM) algorithm, we performed a logarithmic transformation on the data. The purpose of this step was to transform the data into a form that is more consistent with the normal distribution, thereby improving the performance and ACC of the EM algorithm when imputing missing values.

Subsequently, the EM algorithm was used. The EM algorithm is a statistical technique commonly used to deal with missing data, which iterates through the following two main steps: Expectation step (E‐step): Under the current parameter estimation, calculate the expected value of the missing data. Maximization step (M‐step): According to the results of the expectation step, maximize the log‐likelihood function and update the parameter estimates.

After the EM algorithm processing was completed, we performed an inverse logarithmic transformation on the data, restoring the data to its original proportion. This step ensures that the data maintains its original characteristics in subsequent analysis and modeling.

Through logarithmic transformation and the EM algorithm, we can restore missing data to a certain extent, thereby improving the completeness of the data and the ACC of the model.

#### 2.2.4. Different Range Numerical Processing

For features in the dataset with different scales, especially those with different sizes or units, it is necessary to standardize them so that these features can be effectively compared and weighted in the model. Using StandardScaler for standardization processing is a common method. StandardScaler standardizes the data by converting it into a standard normal distribution with a mean of 0 and a standard deviation of 1.

The main advantage of this method is that it converts data with different dimensions to a unified scale, making all features contribute similarly to the loss function in the ML model, thus avoiding model training instability or slow optimization convergence caused by differences in feature value dimensions. In addition, StandardScaler can improve model performance in most cases, especially when using gradient descent optimization algorithms. This standardization method of StandardScaler effectively solves the problems caused by different scale features, ensuring fairness and effectiveness of each feature in the model training process.

In summary, StandardScaler effectively solves the problems caused by different scale features through mean and standard deviation standardization processing, ensuring fairness and effectiveness of each feature in the model training process.

#### 2.2.5. Dataset Sample Imbalance Processing

If there is sample imbalance in the training and validation sets during cross‐validation, it can be solved by oversampling. In this study, the target feature “BMI encoding” has a proportion of 0 (70%) and 1 (30%). Oversampling is to analyze the minority class samples in each cross‐validation fold and then add artificially synthesized new samples to the dataset based on the minority class samples. This is mainly achieved through SMOTE. SMOTE generates a random point from the minority class samples, and its neighboring samples are also included in the construction of new samples for calculation. SMOTE effectively solves the problem of dataset sample imbalance by generating new minority class samples, thereby improving the model’s generalization performance and recognition ability for minority class samples.

### 2.3. Cross‐Validation

In this study, we used repeated stratified K‐fold cross‐validation to evaluate the performance of the prediction model. Specifically, we used five‐fold cross‐validation and repeated it 10 times.

#### 2.3.1. Cross‐Validation Scheme Explanation

Stratified K‐fold cross‐validation: Stratified K‐fold cross‐validation ensures that the proportion of each category in each fold is the same as in the original dataset. For classification problems, especially when there is category imbalance, this method can provide more reliable model evaluation results.

#### 2.3.2. Fold Cross‐Validation (Five‐Fold Cross‐Validation)

The dataset is randomly divided into five mutually exclusive subsets (folds). In each iteration, four subsets are used to train the model, and the remaining one subset is used to validate the model. In this way, we can evaluate the model multiple times without wasting data, thereby reducing the fluctuation in evaluation results caused by different data divisions.

#### 2.3.3. Repeat 10 Times (10 Repeats)

To further improve the stability and reliability of the evaluation, we repeated the entire five‐fold cross‐validation process 10 times. Each repetition will have a different division of the dataset. In this way, we can further smooth the random fluctuations brought by a single division and obtain a more robust evaluation result.

Using the above repeated stratified cross‐validation method, we can make full use of the data to obtain the average performance evaluation of the model under different data divisions, ensuring the generalization ability of the model and the reliability of the evaluation results.

### 2.4. Prediction Algorithm Construction

To reduce the impact of method bias and improve prediction ACC, we considered using three different prediction model algorithms, namely logistic regression classifier, random forest classifier, and gradient boosting classifier. Each model will be evaluated and compared in detail in subsequent experiments to fully understand its performance on the current research problem.

#### 2.4.1. Classifiers

##### 2.4.1.1. Logistic Regression Classifier (Logistic Regression)

The logistic regression classifier is a general linear model commonly used for binary classification problems. It models the log‐odds function and adjusts model parameters by maximizing likelihood estimation. The advantage of logistic regression is its simplicity and interpretability, and it can directly provide the importance weight of features. Although the model is relatively simple, it performs well in many practical applications.

##### 2.4.1.2. Random Forest Classifier (Random Forest)

The random forest classifier is an ensemble learning method that improves classification performance by constructing multiple decision trees and combining their prediction results. Random forest enhances the robustness and generalization ability of the model by introducing randomness (such as randomly selecting features and samples). It can not only handle high‐dimensional data but also effectively deal with overfitting problems, while providing feature importance ranking.

##### 2.4.1.3. Gradient Boosting Classifier (Gradient Boosting)

The gradient boosting classifier is an iterative ensemble learning algorithm that improves overall model performance by constructing a series of weak classifiers (usually decision trees) and optimizing their combination. The gradient boosting method improves the ACC and generalization ability of the model by gradually reducing the model’s prediction error. It performs particularly well in handling complex data and capturing nonlinear relationships and is widely used in various classification and regression tasks.

### 2.5. Performance Evaluation

In this study, we used the confusion matrix analysis as the main tool to evaluate the performance of the proposed model. The confusion matrix is a two‐dimensional matrix that compares the relationship between the model’s predicted results and the actual observed values. Through the confusion matrix, we can calculate multiple performance indicators, including but not limited to ACC, AUC, precision, recall, SPE, *F*1 score, balanced ACC, positive predictive value (PPV), negative predictive value (NPV), etc.

#### 2.5.1. ACC

ACC refers to the proportion of samples correctly predicted by the model to the total number of samples, and the calculation formula is as follows: ACC = (TP + TN)/(TP + TN + FP + FN), where TP represents true positives, TN represents true negatives, FP represents false positives, and FN represents false negatives.

#### 2.5.2. AUC

The AUC is a commonly used indicator to evaluate the performance of binary classification models. The closer the AUC value is to 1, the stronger the model’s ability to distinguish between positive and negative samples.

#### 2.5.3. Precision

Precision (PPV) refers to the proportion of actual positive samples among all samples predicted by the model as positive, and the calculation formula is as follows: Precision = TP/(TP + FP).

#### 2.5.4. Recall

Recall (SEN), also known as sensitivity, indicates the proportion of actual positive samples that the model successfully predicts as positive, and the calculation formula is as follows: Recall = TP/(TP + FN).

#### 2.5.5. SPE

SPE indicates the proportion of actual negative samples that the model successfully predicts as negative, and the calculation formula is as follows: SPE = TN/(TN + FP).

#### 2.5.6. *F*1 Score

The *F*1 Score is the harmonic mean of precision and recall, measuring the performance of classification models in dealing with unbalanced datasets, and the calculation formula is as follows: *F*1 score = 2 × (precision × recall)/(precision + recall).

#### 2.5.7. Balanced ACC

Balanced ACC is the simple average of sensitivity and SPE, and the calculation formula is as follows: Balanced ACC = (sensitivity + SPE)/2.

#### 2.5.8. PPV

The PPV refers to the proportion of actual positive samples among all samples predicted by the model as positive, and the calculation formula is as follows: PPV = TP/(TP + FP).

#### 2.5.9. NPV

The NPV refers to the proportion of actual negative samples among all samples predicted by the model as negative, and the calculation formula is as follows: NPV = TN/(TN + FN).

Through the comprehensive analysis of the above performance indicators, we can fully evaluate the performance of each classification model and then select the most suitable model for predicting MASLD patients with normal BMI.

Through the comprehensive analysis of these performance indicators, we can fully understand the performance of the model in different aspects and effectively evaluate its feasibility in practical applications.

## 3. Results

### 3.1. Characteristics of the Study Subjects

After data preprocessing, a total of 2624 patient data were input into the model, all of whom were nonalcoholic fatty liver disease (NAFLD) (MASLD) patients. The dataset included 52 MASLD‐related feature variables. Among them, 1848 patients had a label of 0 (BMI over 24 kg/m^2^), and 776 patients had a label of 1 (BMI between 18 and 24 kg/m^2^). The target feature column name is “BMI encoding,” including 0 (70%) and 1 (30%) label results.

### 3.2. Constructing the Prediction Model

**Figure 1:**
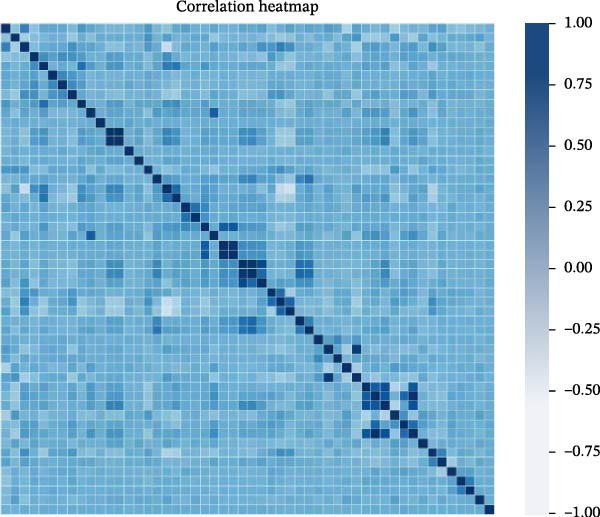
Data correlation heatmap. The correlation values of 1 and −1 indicate a 100% linear and antilinear relationship between two features, respectively. Feature pairs with a correlation value close to 0 are considered nonredundant.

**Figure 2:**
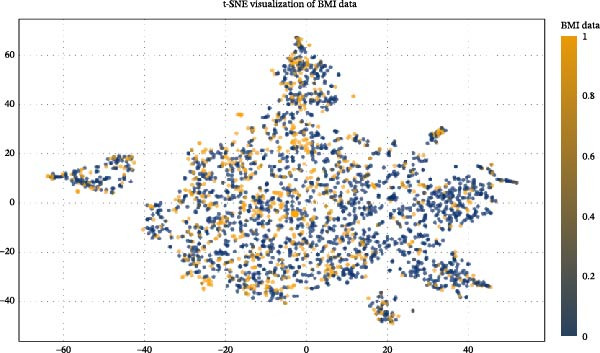
Data visualization using t‐distributed stochastic neighbor embedding (t‐SNE). The t‐distributed stochastic neighbor embedding (t‐SNE) view of the data. Samples are colored according to their respective label results.

Recognizing that combining multiple feature variables in DOU diagnosis is more effective than relying on a single indicator, this study aims to use ML technology to evaluate the diagnostic ability of different feature indicator combinations. We first used t‐SNE diagrams to check the two‐dimensional distribution of various indicators in the training dataset and validation set, which represents the potential of various indicator combinations in the diagnostic process. Further, we analyzed the correlation between various indicators in the training set. The results confirmed that some indicators have significant positive or negative correlations, indicating the joint or antagonistic role of these indicators in diagnostic applications. Data visualization was achieved using t‐SNE, and t‐SNE diagrams were used to check the two‐dimensional distribution of various indicators in the training dataset and validation set to identify potential patterns and structures. Ten‐repeated five‐fold cross‐validation was used for model optimization and feature selection. The prediction model was constructed using a confusion matrix. Three optimal prediction models with the minimum error, random forest, logistic regression, and gradient boosting, were established. Figures 1 and 2 show detailed information.

### 3.3. Model Performance Evaluation

In this study, we selected the optimal algorithm from logistic regression, random forest, and gradient boosting, three ML classification algorithms, through indicators such as AUC, ACC, sensitivity (Recall), PPV, NPV, and *F*1 score.

**Figure 3:**
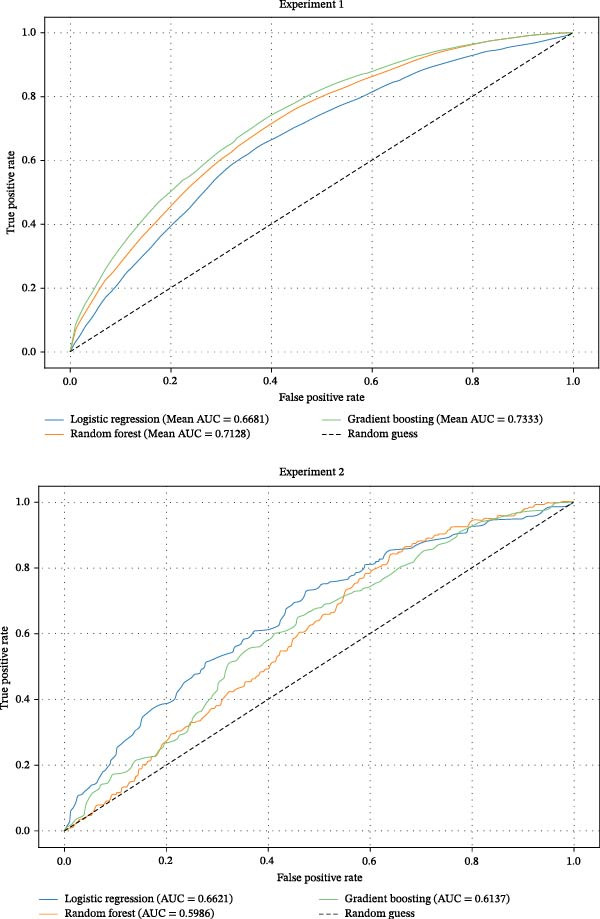
Experiment 1, the average ROC curve comparison diagram of each model for 50 times cross‐validation. Experiment 2 dataset model ROC curve comparison diagram.

The evaluation and analysis of model performance metrics were divided into two experiments:

1. Experiment 1: The final dataset after data preprocessing and feature selection as shown in Table 1 included data from 2624 patients, using a 10‐times repeated five‐fold stratified cross‐validation. As shown in Figure 3, the AUC values of the three classification algorithms range from 0.668 to 0.733, with gradient boosting having the highest AUC. Table 2 shows the average performance indicators and standard deviations of the three classification algorithms under different evaluation criteria. Overall, gradient boosting performed the best in various indicators. Gradient boosting showed excellent performance in many evaluation indicators, especially in AUC and ACC. The standard deviations of the performance indicators of gradient boosting in different cross‐validation folds are shown in parentheses, and the performance indicators of gradient boosting and random forest are relatively stable, indicating strong generalization ability. This suggests that the model can better capture complex patterns and nonlinear relationships in the data and provide more stable and accurate prediction results.
2. Experiment 2: The final dataset from Experiment 1 was used as the training set, and an additional 877 patient data were collected and preprocessed as shown in Table 1, resulting in a total of 699 samples as the external validation set. To further validate the generalization ability of the model, this study evaluated the model performance by external independent datasets. As shown in Figure 3 and Table 2, gradient boosting was still better than the remaining models in terms of ACC (0.65) and SPE (0.80), and its AUC value (0.61) decreased compared with Experiment 1, indicating that the distribution of external data may have an impact on the consistency of model prediction. Logistic regression best performance on AUC (0.66) and NPV (0.80), but the ACC (0.60) and PPV (0.38) were significantly lower. Random forest Is outstanding in SPE (0.83) but significantly weaker than other models in sensitivity correlation measures (*F*1 score 0.26).

**Table 2:** The performance of various models in Experiments 1 and 2.

Experiment	Model	Accuracy	AUC	Precision	Specificity	*F*1 score	PPV	NPV
Experiment 1	Logistic regression	0.63 (±0.0158)	0.67 (±0.0167)	0.42 (±0.0164)	0.63 (±0.0231)	0.51 (±0.0191)	0.42 (±0.0164)	0.81 (±0.0147)
	Random forest	0.72 (±0.0114)	0.71 (±0.0175)	0.53 (±0.0344)	0.88 (±0.0164)	0.40 (±0.0288)	0.53 (±0.0344)	0.76 (±0.0077)
	Gradient boosting	0.73 (±0.0129)	0.73 (±0.0136)	0.55 (±0.0334)	0.87 (±0.0167)	0.46 (±0.0274)	0.55 (±0.0334)	0.77 (±0.0093)
Experiment 2	Logistic regression	0.60	0.66	0.38	0.60	0.48	0.38	0.80
	Random forest	0.65	0.59	0.34	0.83	0.26	0.34	0.72
	Gradient boosting	0.65	0.61	0.35	0.80	0.31	0.35	0.73

Through overall comparison, gradient boosting was considered the best model for predicting patients with normal BMI and fatty liver disease in this study. The performance of the gradient boosting model through cross‐validation was established through confusion matrix analysis. The average AUC was 0.733 (95% CI: 0.7181–0.7485), as shown in Figure 4.

Figure 4(a) Gradient boosting model performance statistics under 50 times cross‐validation, excluding outliers with a standard deviation of ±1.96. ACC: accuracy; AUC: receiver operating characteristic curve under the ROC area; Bal Acc: balanced accuracy; *F*1 Score: *F*1 score; NPV: negative predictive value; PPV: positive predictive value; Precision: positive predictive value; Recall: sensitivity (SNS); Specificity: specificity. (b) The average cross‐validation receiver operating characteristic (ROC) curve of the gradient boosting model.

**Figure:**
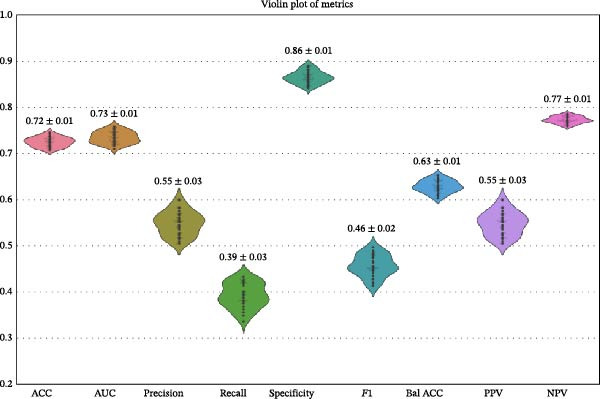
(a)

**Figure:**
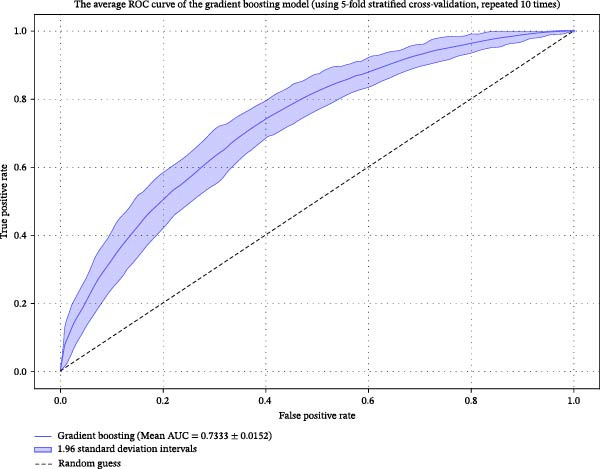
(b)

### 3.4. Feature Weighting and Distribution

**Figure 5:**
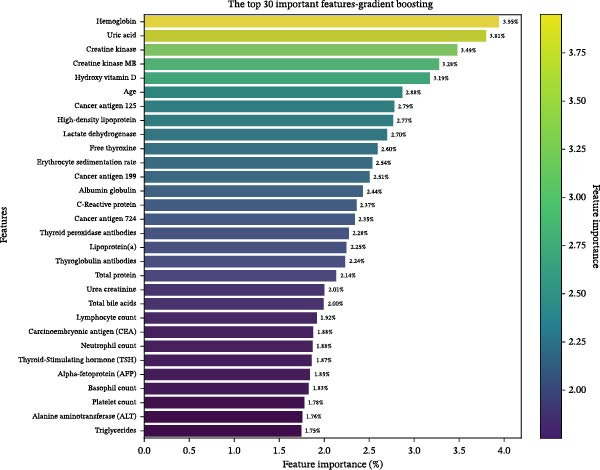
The average feature importance ranking of the gradient boosting model in 50 times cross‐validation folds.

Feature ranking and selection were performed as part of the data preprocessing steps for each fold (Section 2.2). The final feature importance was calculated based on the average of all feature rankings in the MC folds, with the top 30 features listed. The final feature ranking is shown in Figure 5 and Table 3.

**Table 3:** The standard deviation matrix of various performance indicators of the gradient boosting model in 50 times cross‐validation folds.

NAFLD features	Mean	Std	Min	25%	50%	75%	Max
Hemoglobin	3.95%	1.19%	1.18%	3.21%	4.01%	4.85%	5.97%
Uric acid	3.81%	0.68%	2.53%	3.27%	3.68%	4.24%	5.22%
Creatine kinase	3.49%	0.66%	2.29%	3.04%	3.41%	4.07%	5.00%
Creatine kinase MB isoenzyme quantitative	3.28%	0.47%	2.41%	2.95%	3.25%	3.68%	4.22%
Hydroxy vitamin D	3.19%	0.36%	2.42%	2.89%	3.26%	3.50%	3.82%
Age	2.88%	0.56%	1.87%	2.45%	2.93%	3.18%	4.77%
Cancer antigen 125	2.79%	0.52%	1.60%	2.44%	2.78%	3.02%	4.03%
High‐density lipoprotein	2.77%	0.71%	1.68%	2.24%	2.73%	3.16%	4.91%
Lactate dehydrogenase	2.70%	0.40%	1.74%	2.42%	2.71%	3.02%	3.75%
Free thyroxine	2.60%	0.40%	1.90%	2.34%	2.57%	2.85%	3.62%
Erythrocyte sedimentation rate	2.54%	0.37%	1.93%	2.24%	2.61%	2.74%	3.50%
Cancer antigen 199	2.51%	0.52%	1.45%	2.17%	2.44%	2.80%	4.01%
Albumin globulin	2.44%	0.37%	1.70%	2.13%	2.40%	2.67%	3.46%
C‐reactive protein	2.37%	0.42%	1.57%	2.07%	2.42%	2.63%	3.18%
Cancer antigen 724	2.35%	0.36%	1.64%	2.10%	2.28%	2.55%	3.31%
Thyroid peroxidase antibodies	2.28%	0.41%	1.52%	2.02%	2.20%	2.50%	3.52%
Lipoprotein(a)	2.25%	0.37%	1.64%	2.06%	2.16%	2.37%	3.18%
Thyroglobulin antibodies	2.24%	0.32%	1.58%	2.07%	2.29%	2.49%	2.95%
Total protein	2.14%	0.36%	1.38%	1.86%	2.12%	2.34%	3.19%
Urea creatinine	2.01%	0.42%	1.24%	1.73%	1.96%	2.34%	3.31%
Total bile acids	2.00%	0.32%	1.36%	1.78%	2.00%	2.18%	2.90%
Lymphocyte count	1.92%	0.43%	1.13%	1.57%	1.93%	2.22%	2.92%
Carcinoembryonic antigen (CEA)	1.88%	0.29%	1.40%	1.66%	1.86%	2.12%	2.76%
Neutrophil count	1.88%	0.30%	1.12%	1.72%	1.87%	2.08%	2.51%
Thyroid‐stimulating hormone (TSH)	1.87%	0.31%	1.25%	1.60%	1.89%	2.12%	2.33%
Alpha‐fetoprotein (AFP)	1.85%	0.28%	1.26%	1.65%	1.84%	2.02%	2.74%
Basophil count	1.83%	0.52%	0.94%	1.49%	1.83%	2.08%	3.86%
Platelet count	1.78%	0.28%	1.26%	1.56%	1.77%	1.98%	2.38%
Alanine aminotransferase (ALT)	1.76%	0.45%	1.02%	1.53%	1.70%	1.91%	3.58%
Triglycerides	1.75%	0.36%	1.07%	1.48%	1.71%	1.93%	2.63%
Glucose	1.74%	0.33%	1.21%	1.45%	1.74%	2.00%	2.66%
Glutamyl transpeptidase (GGT)	1.71%	0.27%	1.06%	1.56%	1.75%	1.87%	2.22%
Alkaline phosphatase	1.68%	0.27%	1.23%	1.47%	1.67%	1.89%	2.26%
Creatinine	1.66%	0.36%	0.96%	1.41%	1.70%	1.85%	2.69%
Glycated hemoglobin	1.64%	0.32%	1.09%	1.40%	1.58%	1.84%	2.39%
Monocyte count	1.64%	0.31%	0.99%	1.41%	1.58%	1.87%	2.39%
Free triiodothyronine (FT3)	1.61%	0.29%	1.02%	1.41%	1.60%	1.80%	2.36%
Urea	1.52%	0.26%	1.09%	1.33%	1.49%	1.68%	2.34%
Direct bilirubin	1.52%	0.33%	0.82%	1.30%	1.54%	1.73%	2.55%
Cystatin C	1.38%	0.22%	0.95%	1.23%	1.35%	1.56%	1.86%
Albumin	1.36%	0.23%	0.92%	1.21%	1.34%	1.54%	1.95%
Eosinophil count	1.35%	0.23%	0.72%	1.18%	1.35%	1.47%	1.83%
Low‐density lipoprotein	1.30%	0.28%	0.68%	1.15%	1.27%	1.47%	1.94%
Aspartate aminotransferase (AST)	1.22%	0.23%	0.78%	1.06%	1.20%	1.38%	1.84%
Indirect bilirubin	1.22%	0.24%	0.70%	1.05%	1.24%	1.38%	1.86%
Total cholesterol high‐density lipoprotein	1.15%	0.26%	0.67%	1.00%	1.13%	1.31%	1.94%
Total bilirubin	1.11%	0.26%	0.53%	0.91%	1.11%	1.25%	1.76%
Total cholesterol	0.95%	0.18%	0.63%	0.83%	0.93%	1.07%	1.44%
Non‐HDL cholesterol	0.88%	0.23%	0.53%	0.69%	0.82%	1.04%	1.44%
Gender	0.25%	0.11%	0.08%	0.17%	0.25%	0.29%	0.53%

## 4. Discussion

The prevalence of MASLD in the general BMI population is ~5%–20%. Studies have indicated that the proportion of MASLD patients with normal BMI in Asian populations is higher than in Western populations, and among those with normal BMI type 2 diabetes, the prevalence of MASLD can be as high as 20%–30%. This may be related to the visceral fat accumulation characteristic of Asian populations [20, 21]. Research has found that hemoglobin concentration may be associated with an increased risk of MASLD and the progression of liver fibrosis. High levels of hemoglobin are closely linked to insulin resistance in diabetic patients, which is one of the core mechanisms of MASLD [22, 23]. Numerous studies have reported on the relationship between serum uric acid and MASLD [24, 25]. This correlation may be mediated by insulin resistance. High uric acid may play a role in the reduction of NO bioavailability, leading to a decrease in the supply of NO from endothelial cells and inducing insulin resistance [26, 27]. Studies have shown that individuals with higher CK levels are more likely to suffer from metabolic syndrome. The possible mechanisms include abnormal muscle metabolism, insulin resistance, chronic inflammation, oxidative stress, and muscle injury [28–30]. Studies have indicated that vitamin D deficiency is more prevalent among patients with MASLD, and it exhibits a negative correlation with the severity of MASLD [31, 32]. Some studies suggest that vitamin D can reduce liver inflammation and oxidative stress by inhibiting the p53‐p21 signaling pathway and related cellular senescence [33], decreasing Toll‐like receptor expression [34], inhibiting inositol protein, promoting nuclear translocation of nuclear factor erythroid 2‐related factor 2 (NFE2L2) [35], and improving hepatic insulin resistance through the activation of hepatocyte nuclear factor 4α (HNF 4α) mediated by VDR [36], thereby improving metabolic syndrome.

In this study, we identified the top 30 predictive features based on their average importance across cross‐validation folds. Hemoglobin, uric acid, creatine kinase, creatine kinase isoenzyme, and vitamin D emerged as the most influential variables, consistent with prior studies linking these biomarkers to MASLD pathogenesis.

Among the three ML models evaluated—random forest, logistic regression, and gradient boosting—the gradient boosting model demonstrated the best overall predictive performance, achieving an average AUC of 0.733 (95% CI: 0.7089–0.7578) and outperforming the others across multiple metrics (Table 2). Its superiority was particularly evident in AUC (0.733 vs. 0.71 for random forest and 0.67 for logistic regression) and sensitivity (0.68 vs. 0.45 and 0.52, respectively). Gradient boosting, an iterative ensemble method that combines weak classifiers (typically decision trees) to optimize predictive ACC, excels at capturing complex nonlinear relationships in high‐dimensional data. This capability, combined with its stability across repeated cross‐validations, supports its selection as the optimal model for identifying lean MASLD patients at elevated metabolic risk. By leveraging routine laboratory parameters, this model can assist clinicians in risk stratification and personalized management of diabetic patients with normal BMI, potentially improving long‐term outcomes.

Nevertheless, this study has several limitations. First, the retrospective design and reliance on real‐world data introduce potential bias from missing values, despite rigorous imputation using the EM algorithm. Second, the absence of imaging‐negative diabetic controls precludes estimation of true SPE; therefore, our model should not be used to diagnose MASLD in the general diabetic population—it is intended only for risk stratification among patients already diagnosed with MASLD. Third, histological staging was unavailable, limiting our ability to link predictions to fibrosis severity. Fourth, the lack of multicenter prospective cohorts restricts assessment of generalizability across diverse populations. Finally, only routine laboratory tests and basic demographics were included; multimodal data (e.g., imaging, genetic markers) could further enhance model performance.

## Funding

No funding was received.

## Disclosure

The study was retrospective study.

## Conflicts of Interest

The authors declare no conflicts of interest.

## Data Availability

The data that support the findings of this study are available on request from the corresponding author. The data are not publicly available due to privacy or ethical restrictions.Yuelan
